# Norwegian nursing students' experience during clinical placement in an African country: Communication, relationship building and nursing identity. A qualitative study

**DOI:** 10.1002/nop2.1440

**Published:** 2022-10-27

**Authors:** Tove Kristin Greaker, Solveig Kirsti Grudt, Ingvild Aune

**Affiliations:** ^1^ Department of Public Health and Nursing, Faculty of Medicine and Health Sciences Norwegian University of Sciences and Technology Trondheim Norway; ^2^ Department of Clinical and Molecular Medicine, Faculty of Medicine and Health Sciences Norwegian University of Sciences and Technology Trondheim Norway; ^3^ Faculty of Health Sciences UiT‐The Arctic University of Norway Tromsø Norway

**Keywords:** communication, cultural sensitivity, identity, learning through practice, nursing education, relationship

## Abstract

**Aim:**

The aim of this study was to gain a deeper understanding of the experience of Norwegian bachelor nursing students during clinical placement in an African country, with a focus on communication, relationship building and nurse identity.

**Design:**

Explorative, qualitative methods were used.

**Methods:**

The data consisted of individual written reflection notes from 8 students' clinical placement in Africa, and transcripts from one semi‐structured focus group interview. The materials were analysed with systematic text condensation.

**Results:**

The students described their experience with the community of practice as challenging and enlightening. They found themselves in contexts where communication and language problems occurred. The students described how important relationships were for their practical training, and how this helped shape their nursing identity.

## 1 INTRODUCTION

In hospitals and the municipal health services in Norway, both nursing students and fully employed nurses play a crucial role when implementing their profession. Nursing as a profession aims at fulfilling society's requirement for sufficient health services that cover all citizens. A health professional needs both knowledge and action skills to cope with a number of situations (Irgens, 2007, pp. 20–30). The training of nurses takes place both at higher education institutions and locations of clinical placement. The European Union's directives for skills development make certain requirements regarding the clinical placement venues. The nursing education observes the EU guidelines, which demand learning situations that, for instance, offer the opportunity to develop relational skills (Henriksen et al., 2020). According to Wenger (2004), communities of practice enable identity building, as we let ourselves be influenced by others while we influence other people. Our identity is based on our ability to create meaning that accentuates our social environment and sense of belonging to various social contexts. Human beings are social creatures that craft their identity based on relations to communities of practice (Wenger, 2004). According to Rasmussen et al. (2018), the professional nursing identity is characterized by three main components: Who am I? What am I doing? And what context do I find myself in? The development of identity is dependent on our context. Kristoffersen (2021) claims that the professional identity is marked by the implementation of professional responsibility, traditions, as well as the functions and values of the relevant professional field. Such an identity requires commitment, competence, responsibility, and decisions based on clear values. The use of a professional language emphasizes the professional role and assists the development of a nursing identity (Thompson & McNamara, 2021).

## 2 BACKGROUND

A number of study programmes in nursing have sent students to lower and medium income countries for international clinical placement. Studies indicate that placements abroad lead to a higher understanding, knowledge about other countries and disease panorama, ethical challenges, culturally sensitive interaction and a stronger identity (Grudt & Hadders, 2017; Huffman et al., 2020; Jørgensen & Hadders, 2015; Philips et al., 2017).

Communication means to be linked to someone and implement something collectively. Relational communication is marked by an exchange of words and non‐verbal signs between two or more individuals. In the encounter with another person, there will always be dissimilarity involved (Eide & Eide, 2017, pp. 17–40). Students on placement abroad have been challenged in areas such as role expectations, culturally sensitive interaction and communication, including language and accents (Chulach & Gagnon, 2016; Henderson et al., 2016; Meaux et al., 2021; Repo et al., 2017). The establishment of positive relations between supervisors and nurses contributes to better learning outcomes for students on placement (Rebeiro et al., 2021). However, challenges tend to occur when health professionals from different countries and cultures meet in a complex, inter‐cultural setting marked by different approaches to problem solving (Tingvold & Munkejord, 2021; Pawlak, 2021).

The right of patients to receive responsible nursing services is protected by nurses across the world and officially adopted by The International Council of Nurses. The ethical guidelines have been reviewed and now include the ethical ideals of global health (ICN, 2021). A study by Gilliland et al. (2016) focuses on nursing integrity and how exchange students get the experience of being confronted by their own values and prejudices in a different society, and also how they come across routines of care and treatment that are different from what they are used to. The experience of having your own values challenged is highlighted by Meaux et al. (2021). Rasmussen et al. (2021) point out that well‐functioning communities enable you to improve your self‐understanding, self‐respect and self‐awareness. Values like trust, justice and responsibility are also beneficial.

Lave & Wenger (2007, p. 102) use the term «legitimate peripheral participation» to describe contexts where the participants are not yet fully integrated into or accepted as members of a community of practice. To change the content of one's role is a time‐consuming process that demands communication, relationship building and interaction. As members of a community of practice, we are able to present our attitudes and take part in collective action.

### 2.1 Aim

The aim of this study was to gain a deeper understanding of Norwegian nursing students' experience during clinical placements in an African country, focusing on how the meaning of communication and relationships may affect nursing identity.

## 3 METHOD

### 3.1 Study design

The study had an exploratory design. Eight bachelor's degree nursing students completed 12 weeks of clinical placement in a country in Africa, as part of the final semester of their bachelor's degree. All the students who went to Africa participated in the study. During their theoretical studies, the students had acquired knowledge about ethics, communication, cultural sensitivity and interaction. Each student went through a briefing session before departure and a debriefing session along with their supervisor and fellow students upon their return.

### 3.2 Ethical considerations

Approval of the study was obtained from the Norwegian Centre for Research Data, ID820936 (NSD). The students were given written information describing the purpose of the study, their own rights and the principles of confidentiality. Before the focus group interview was initiated, the students were reminded of the content of the written information. To ensure the anonymity of the participants, the transcripts from the interviews were coded. All data material was handled in accordance with established guidelines of confidentiality. Participants were approached by email. There were no personal bonds or conflicts of interest between the informants and interviewers. The exact name of the country where the study was performed was anonymised to a country in Africa.

### 3.3 Data collection

A qualitative approach was selected for the data collection. As part of the clinical placement, each student wrote two reflection notes that contained 700 and 2,500 words, respectively. These focused on learning objectives, expectations and reflections linked to situations of learning during clinical placement. In addition, the students were invited to participate in a focus group interview 6 months after returning back home. Prior to the interviews, a semi‐structured interview guide with open‐ended questions was developed. The guide was used rather as a tool for data collection than a checklist (Table 1). The students submitted their reflection notes, and all of them participated in a focus group interview that lasted 90 min. The focus group interview was recorded on a tape designed and recommended for research purposes. Two of the researchers participated as moderator and secretary. In this study, we wanted to focus on both the individual experiences reflected in the students' assignments and their opportunity to discuss and express their different experience with each other. Malterud (2012a, pp. 20–21) claims that focus interviews can provide insight into a phenomenon. The focus group interviews have a more narrow scope than individual interviews. The informants have less time to speak in focus groups, so the possibility of validating information during the interview is more limited. According to Malterud (2012a, pp. 132–133), identification of and variation within a topic in focus group interviews can straighten the intern validity. As researchers, we assume that supplementing the focus groups with individual written reflection notes from the clinical placement could accentuate the students' perspectives in the data material. Malterud (2012a, pp. 136–137) claims that group discussions where participants share their experiences could generate further scientific knowledge. As researchers, we try to understand the text from the informant's point of view. The data collection took place from January 2019–December 2019 at workplace.

**TABLE 1:** Interview guide

Tell a bit about your experience from the clinical placement abroad What did you find positive/negative during clinical placement? Which values of the nursing profession were affected during clinical placement? Which dilemmas/problems (if any) occurred during clinical placement? What did you perceive as the reasons for dilemmas that occurred? What could you do to handle dilemmas/problems that occurred? How did you experience being in such situations? ‐‐‐‐‐‐‐‐‐‐‐‐‐‐‐‐‐‐‐‐‐‐‐‐‐‐‐‐‐‐‐‐‐‐‐‐‐‐‐‐‐‐‐‐‐‐‐‐‐‐‐‐‐‐‐‐‐‐‐‐‐‐‐‐‐‐‐‐‐‐‐‐‐‐‐‐‐‐‐‐‐‐‐‐‐‐‐‐‐‐ How could your experiences benefit your future work as a nurse? What have you experienced in a professional context following your return back home? What learning outcomes do you find most useful for your work as a nurse?

### 3.4 Data analysis

The interview was transcribed by one of the moderators. The text from the reflection notes supplemented the interview transcription. The analysis was based on systematic text condensation and a modified version of Giorgi's phenomenological analysis (Malterud, 2017). Systematic texts condensation is useful method of analysis for both transcriptions and written texts (Malterud, 2012b). The process of analysis involves four steps: Putting aside one's pre‐conceptions, the complete material was studied to gain a general impression. Both researchers agreed on the overarching themes. The material was then reviewed sentence by sentence before it was condensed and finally re‐contextualized based on quotations from the participants. Quotations from the focus interview were marked with I, while quotations from the students' reflection notes were marked with RN (respondent 1–8). Two main themes emerged from the analysis: language challenges and relational development versus nursing identity (Table 2). The COREQ checklist was used for actual questions (Tong et al., 2007).

**TABLE 2:** Example of the analysis process

Meaningful units	Codes	Subtheme	Main theme
“What became increasingly clear was the importance of maintaining good relations with the regular employees at our ward. This greatly affected how much we were able to influence things. And in cases where we enjoyed good relations, we were allowed to carry out distraction techniques with children”	Content of relations Influence strengthens the students autonomy Relations as a gatekeeper for nursing practice Being part of the employees	To meet the students need for clinical practical learning	Relational development versus nursing identity

## 4 RESULTS

Based on the analysis, two main themes were identified: language challenges and relational development versus nursing identity.

### 4.1 Language challenges

#### 4.1.1 Verbal and non‐verbal communication

In the country where this study was performed, English is the main language. In addition, there are about 9 tribal languages, of which two are the most common. Most of the conversations in this actual country were held in English. According to the students, it was at times exhausting to communicate. In many cases, the English used was heavily accented and the nurses often spoke in a low voice. They found that this caused a growing distance between the regular employees, themselves and the patients. The students often felt superfluous and ignored. The language barrier led to misunderstandings and made dialogue and interaction challenging. The regular employees were used to switching between different languages, and the students had a feeling of being discussed in local languages, which made them feel anxious and insecure. After a while they found that the best way to cope with such situations was to make eye contact with the staff and respond with self‐deprecating humour.

RN4: «…After a while one becomes familiar with the cultural code and learns how to respond. It seems we were more included when daring to be a bit a cheeky and bold, like the others».

The students encountered patients and family members who were not able to read and write. To establish a common dialogue and contact was challenging, as patients tended to speak broken English and preferred to use tribal languages. This often turned the students into observers rather than active participants. Local employees were used as interpreters, though the students noticed that not everything was translated. The students decided to learn some words and phrases of the tribal languages. These words became icebreakers in establishing trust and contact. *I: «To know some phrases in a different language is a good way of building relations»*. Moreover, the students used body language and non‐verbal ways of communicating in an attempt to create a common understanding. Some students worried that body language and cultural codes could cause misunderstandings about the emotional state of the patients. Was the patient sad, depressed or angry? The students decided to be patient. Eventually, they became more familiar with the cultural codes were thus able to understand body language better. The students found the language barriers to be a valuable experience.

RN7: «It was challenging, but also enriching to be forced to use body language, drawings and simplified expressions to ensure that patients understood what you were trying to say».

### 4.2 Relational development versus nursing identity

#### 4.2.1 Establishing contact

The first meeting with the venue of clinical placement was described as a culture shock. *I: «Some of the difficulties we experienced came as a result of the meeting with a different culture»*. In addition, few of the local employees had been prepared for the arrival of the students. The students needed to explain who they were, where they came from and why they were there. The students described this country's health system as rigidly organized, with the students themselves far down in the hierarchy. According to one student: *I: « …I have experienced to be left alone, receiving both praise and rebuke in the attempt to discover my own role in a culture far from home»*. It was easy to observe the different roles in the system. For instance, doctors and unskilled health workers wore different uniforms. The students came across a variety of people who regardless of their economic resources willingly shared their belongings. In spite of this, the students experienced clear cultural differences that tested their student role and understanding of their professional identity.

I: «…I feel I have been through a great personal development, ….I have learned a lot about patience…. learned a lot about myself, the differences of human beings and cultures, and found new ways to handle things…».

The students worked to create relationships based on mutual understanding. To be an outsider in terms of language was a new experience. They agreed not to speak Norwegian among themselves whenever local people were present. It was demanding to find good ways of establishing contacts. The students described situations where they forced themselves to remain calm to avoid behaving in ways that could be interpreted as offensive. There was initially a fine balance between initiative and a humble approach. To show interest and alertness seemed like a wise approach.

I: « …the others get the impression that we at least are willing to give it a try. This could be a first step towards getting to know each other better».

#### 4.2.2 Contact with patients

The students described relationship building as a time‐consuming process. With time, they became more familiar with the regular employees. The students also made an effort to build positive relations with the patients. From a dialogue with a young boy: *RN3: «…I let the boy lead the conversation so that he could talk about the things he liked…»*. They emphasized qualities like understanding, cooperation and accepting differences as important when establishing contacts. They felt that the patients were often deprived of social contact, as implementation of practical procedures was given priority at the expense of building relationships. There were generally few earnest conversations with the patients, whose need for privacy and tranquillity was not emphasized. Conversations about patients could happen everywhere and active listening was not a familiar concept:

I: «When meeting children and psychiatric patients who did not necessarily have the same understanding of things as us, I often tried to build relationships. Even if I would not succeed, I felt I owed it to the patient to give it a try».

One student described a situation where the patient did not understand what the staff member was asking. She found it hard to intervene in the conversation:

RN5: «In my mind, the doctor initially could have spent some time to put the patient at ease. He could have presented himself and explained what he would do, while adjusting the words and tone to the patient's age and situation».

#### 4.2.3 Students' learning

To be noticed and respected in a professional sense was a demanding process, according to the students. To establish contact with the regular employees was critical in the attempt to get included in relevant work tasks. Having passed this barrier, it was easier to achieve more interaction. This enabled the students to ask questions more freely and get answers to things they wondered about. They were eventually able to participate more actively and present their own ideas. For instance, they used soap bubbles as a distraction technique to alleviate pain among children. According to the students, children are more likely to react with fear towards uniformed nurses. Building relations with children was not a priority at the hospital.

I: «What became increasingly clear was the importance of maintaining good relations with the regular employees at our ward. This greatly affected how much we were able to influence things. And in cases where we enjoyed good relations, we were allowed to carry out distraction techniques with children».

The students described their wishes and expectations towards their clinical placement. Their objectives included learning different disease panorama, procedures, cultural sensitivity, communication and language – all ingredients in their training in professional nursing. A number of the students found that their professional perspective was reinforced through the stay abroad and they perceived a strong unity in their group:

RN7: «I felt that even if the placement in NN was marked by bureaucracy and cultural differences, I have learned a lot about myself, my good qualities and not so good ones, and realized what kind of nurse I want to be».

## 5 DISCUSSION

### 5.1 Communication, building relationships and development of nursing identity during clinical placement

The students arrived in an African country to learn about nursing. However, it soon proved difficult to do things the way they expected or to be trusted with relevant professional tasks. The students arrived as «legitimate peripheral participants» (Lave & Wenger, 2007). They assumed the role of outsiders in a hierarchical structure and needed to operate in a system with a clear understanding of rank and position. Their role needed to be clarified in their meeting with the local employees. According to Eide and Eide (2017), a role is based on an accumulation of the formal, but also informal expectations we encounter. It was early on challenging for the students to fulfil the expectations of others while satisfying their own. To establish trust was a key challenge. Eide and Eide (2017) claim that when we relinquish control, others will step in to take charge. To show trust represents an ethical challenge, as we may decide to reject, listen to, assist or bypass the other party. Relationships within the health system often tend to be asymmetrical. Chulach and Gagnon (2016) describe a power‐centred hegemony in the health services. Nurses tend to have a subordinate position compared to the doctors. Roles and identity are shaped by the organization and the positions of the employees. In cases where professional, cultural systems collide, the result can be a dialogue that produces a new common understanding and discourse. According to Rebeiro et al. (2021), professional language may contribute to a clarification of roles and reinforce the professional identity.

In this study, the students emphasized language barriers in both a verbal and non‐verbal sense. They tried to get to know their hosts, who they described as open‐minded and sociable people who gave them a warm welcome, even if conversations did not happen on the students' terms. As they learned to know the employees better, they found it easier to take initiatives and be trusted with relevant work tasks. Both Grudt and Hadders (2017) and Jørgensen and Hadders (2015) described students who experienced a chaotic and emotionally exhausting placement abroad, where language problems proved an obstacle against interaction and inclusion. On the other hand, to be in a community of practice abroad reinforces the sense of unity within the student group.

Henderson et al. (2016) stresses how learning to know new peoples is important for your cultural understanding. To remain a bit reserved and wait until being addressed by the local staff members proved a wise strategy. To be too pushy at an early stage could make it more difficult to build relationships later.

To get fully integrated was not an easy task. The students mentioned language barriers as a hindrance against satisfactory communication, which in turn caused relational distance. This is supported by other studies, which emphasize cultural sensitivity and language problems as a key challenge (Huffman et al., 2020; Meaux et al., 2021). In spite of this, there are always opportunities for learning and understanding in the encounter with foreign languages. The students in this case learned a bit of tribal languages and came to realize the importance of cultural skills. This enabled more opportunities for establishing contacts and gaining access to the community of practice. According to Mikkonen et al. (2020), expectations towards one's student role and positive relations to the supervisor are valuable, as firmly established bonds favour the chances of learning. However, Hultsjö et al. (2019) stress that both theoretical and practical training in cultural awareness are needed.

In the process of entering a community of practice, the establishment of trust, building relations and taking responsibility for one's learning were all gradually reinforced. Nevertheless, the students' ethical standards and attitudes were challenged. There are obvious differences between the Norwegian and the health care systems in the country where this study was performed, and the students felt a need to preserve their professional integrity. A study by Tingvold and Munkejord (2021) highlights the importance of regular clarification meetings for a culturally diverse group of staff, given obvious differences regarding opinions, solutions and problem solving. Pawlak (2021) stresses how Norwegian and Polish nurses approach their profession differently. To get an insight into the ways of listening, discussing and behaving within other cultures is crucial in developing awareness about one's own professional identity and approach to nursing.

The development and implementation of relational skills are vital for nursing, both nationally and globally. This does not only benefit communication within the framework of responsible nursing in general (Kwame & Petrucka, 2020; Litland & Rommetveit, 2018; Repo et al., 2017; Wune et al., 2020), but also enables training in active listening and the development of empathy (Haley et al., 2017), in addition to the ability to maintain values and good attitudes in the patient–nurse relationship (Rider et al., 2014). ICN (2021) provides clear guidelines for value‐based behaviour and decision making. Kristoffersen (2021, pp. 342–344) emphasizes commitment, responsibility, and ethical awareness as key components of a professional nursing identity.

The students described an active and inspiring workday. Wenger (2004) claims that dedication is vital to achieve a sense of belonging during clinical placement. But the clinical placement and operating in a unified group are not sufficient factors – a sense of meaning is also needed. Meaning is created through a process and can be negotiated between different parties. The process of working together creates both similarities and differences. It enables you to grasp what is different. In a community of practice, each participant tends to discover his/her unique position. Everyone is able to find their own identity, which is both integrated and defined through dedicated activity. The participants are able to produce new understandings and identities based on the discovery of new perspectives. The building of identity could be considered a dynamic process, where relational skills make up a part. Targeted action in a common sphere of practice may contribute to a collective experience of professional identity (Wenger, 2004). In many ways, this is supported by Rasmussen et al. (2018), who focuses on three factors that are relevant for the creation of a nursing identity. Which context do you operate in? Who are you? And what do you do? The students in this case were moving from «the outside to the inside» in their practical placement in Africa. Communication and the building of relations are key factors when trying to gain access to the place where action is implemented, while developing awareness about one's own values, attitudes and integrity. This, it seems, was significant for the nurses' ability to discover their nursing identity. The students wanted to talk the same language as the others and to identify as «something» or perhaps «someone» when practicing their profession. Their descriptions of the clinical placement reveal how they perceived their own role and how they wished to implement it. A number of learning outcomes were achieved during their stay abroad. Daae‐Qvale (2016) claims that the mandate of health professions is expressed through what she describes as targeted activity. To be someone for somebody else, and to implement practical action, is part of the development of a professional identity.

Henriksen et al. (2020) point out that a number of venues of clinical placement that receive exchange students have not been subject to proper quality assurance. The results from both the current study and previous studies confirm that clinical placement in lower and medium income countries promotes training, learning and awareness about one's professional field (Grudt & Hadders, 2017; Jørgensen & Hadders, 2015; Philips et al., 2017). According to Wenger (2004), there are challenging situations that block access to social contexts. Learning is affected by the action and attitudes we share with other people. A higher degree of participation and involvement could lead to a better understanding of what identifies the interests of a community. Hovland and Johannesen (2021) support the finding that students who completed an international clinical placement experienced personal and professional development.

#### 5.1.1 Practical implication of the results

It seems useful that the lectures responsible for the students going abroad have a close communication with the students concerning different challenges both before, under and after their clinical placement period. It seems that collaboration between lectures in Norway, students, clinical nurses/leaders in the host country, is important.

### 5.2 Limitations

The results are based on the experience of only 8 Norwegian bachelor's degree nursing students, their reflection notes and discussions about learning practice during their twelve weeks in an African country. It is a limited sample and due to the qualitative design of the study, it is hard to claim that the experience of the participants in this study needs to be representative for students in general. The informants talked freely about their experience and offered rich and comprehensive accounts that provide in‐depth information about the topic we have examined.

## 6 CONCLUDING REMARKS

The students encountered barriers related to language and communication when they arrived in this study in an African country. They gradually discovered approaches that enabled them to develop bonds with patients and employees. They experienced that good relationships opened the gates to the community of practice. The students were tested on their sense of commitment and responsibility, their ethical values and the way they wished to implement nursing. These are all key factors in the development of a professional nursing identity.

## AUTHOR CONTRIBUTIONS

**Tove Kristin Greaker:** Developing project plan and interview guide, defining the purpose of the study, submitting study to the Norwegian Centre for Research Data (NSD), data collection, transcription and analysis, literature search and writing the article. **Solveig Grudt:** Project manager, developing interview guide, submitting study to the Norwegian Centre for Research Data (NSD), data collection, participating in analysis, and commenting on article writing. **Ingvild Aune:** Supervisor, quality assurance of analysis and article writing.

## CONFLICT OF INTEREST

None declared.

## CLINICAL TRIAL REGISTRATION

Norwegian Centre for Research Data (NSD): ID 820936.
